# Ginseng Berry Prevents Alcohol-Induced Liver Damage by Improving the Anti-Inflammatory System Damage in Mice and Quality Control of Active Compounds

**DOI:** 10.3390/ijms20143522

**Published:** 2019-07-18

**Authors:** Dae Young Lee, Min-Jee Kim, Dahye Yoon, Young-Seob Lee, Geum-Soog Kim, Yung Choon Yoo

**Affiliations:** 1 Department of Herbal Crop Research, National Institute of Horticultural and Herbal Science, RDA, Eumseong 27709, Korea; 2 Department of Microbiology, College of Medicine, Konyang University, Daejeon 35365, Korea

**Keywords:** ginseng berry, alcohol-induced liver damage, oxidation, anti-inflammation, ginsenosides

## Abstract

The ginseng berry contains a variety of biologically active compounds and has a higher ginsenoside content than its roots. This study focused on the hepatoprotective activity of ginseng berry extract prepared by enzyme treatment (EGB) compared to the non-enzyme-treated ginseng berry extract (GB) and quality control of EGB. The feeding effect of EGB on alcohol-induced liver damage (AILD) was investigated by measuring the serum levels of aspartate aminotransferase (AST) and alanine aminotransferase (ALT) compared with those of EtOH-fed mice. Furthermore, cytokine levels in the culture supernatants of EGB- or GB-treated RAW 264.7 cells were determined by enzyme-linked immunosorbent assay. The developed method was applied to the simultaneous quantification of four major ginsenosides in EGB using UPLC-QTOF/MS. Treatment with EGB at a dose of 0.5 or 1 mg/mouse significantly suppressed the AST and ALT levels in mice with AILD. Enzyme-treated ginseng berry was also found to suppress the production of inflammatory mediators like nitric oxide (NO), tumor-necrosis factor-α (TNF-α), interleukin-6 (IL-6), and prostaglandin E2 (PGE2) in lipopolysaccharide (LPS)-stimulated RAW 264.7 macrophages, showing higher activity than that of GB. The amount of ginsenoside Re, F5, F3, and Rd in the EGB obtained using UPLC-QTOF/MS was 45.9, 3.3, 4.0, and 6.2 mg/g, respectively. These results suggest that EGB has a potential effect on AILD, and its hepatoprotective effect provides beneficial insights into developing new candidates for the prevention and cure of AILD. Also, this study demonstrated the utility of UPLC-QTOF/MS-based major compounds for quality control (QC) of EGB.

## 1. Introduction

Alcohol-induced liver damage (AILD) is recognized as a major cause of liver-associated morbidity and mortality worldwide [1]. Although it is well known that alcohol causes liver damage, the precise mechanisms related to the pathogenesis of AILD are not understood clearly. Many factors play important roles in the progression and pathogenesis of AILD, including gender and ethnic differences, disorder of metabolic function, nutritional condition, immunological actions, and oxidation-related stress [2]. Alcohol consumption has tended to increase all over the world, and has given rise to various health troubles related to functional disorder in the liver [3,4]. Because the liver is the main organ responsible for metabolic function for ingested alcohol, a series of physiological changes in the liver caused by alcohol exposure may leave the organ injured. Thus, the method of detecting biomarkers in the liver and blood has been widely used to diagnose the early stages of liver damage, and measure functional factors for the prevention or cure of chronic alcoholism or hepatic lesions [2,3,5,6,7].

**Figure 1:**
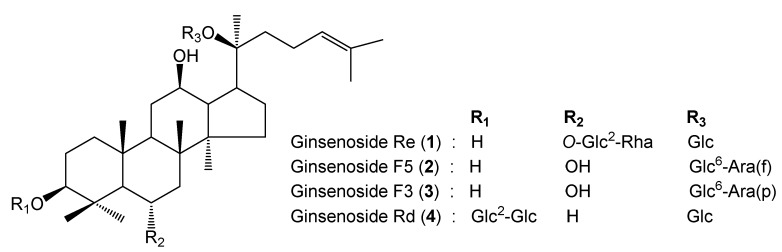
Structure of active compounds of ginseng berry. (Glc: β-D-glucopyranosyl, Ara(f): α-L-arabinofuranosyl, Ara(p): α-L-arabinopyranosyl, Rha: α-L-rhamnopyranosyl).

*Panax ginseng* Meyer, a famous medicinal herbal source in Asia, has been used as a tonic and to treat various diseases [8,9,10,11]. Biologically active ginsenosides (also called ginseng saponins) from the different parts of *Panax ginseng*, including roots, flower buds, leaves, and berries, have been studied extensively, and many triterpenoid glycosides have been isolated as major ingredients [12,13,14]. Interestingly, quantitative analysis revealed that the total ginsenoside content of ginseng berry was over three times higher than that of ginseng roots. Thus, ginseng berries contain a high amount of ginseng saponins, a member of the dammarane-type triterpenoids family. Among these, ginsenoside Re, Rd, F5, and F3 are the key compounds of ginseng berry (Figure 1), showing inhibitory effects on D-GalN/lipopolysaccharide (LPS)-induced liver injury in mice, as well as anti-inflammation and anti-oxidative activity [15,16,17,18]. In particular, ginseng berry extract contains highly concentrated ginsenoside Re 30 times greater than the extract of ginseng root. Therefore, ginseng berry, which has a large amount of ginsenoside Re, is thought to be more effective than ginseng root [19]. Many studies have been conducted to modify the structural composition of ginsenosides to improve their activity using enzyme and heating methods [20,21]. Ginseng is usually processed by methods, such as acid hydrolysis and fermentation, through which major ginsenosides would be transformed into minor ginsenosides [22,23]. However, the effect brought about by the bioconversion of ginsenosides and whether they had an impact on the hepatoprotective activity of the enzyme-treated ginseng berry, as well as the difference between enzyme-treated ginseng berry extract (EGB) and non-enzyme-treated ginseng berry extract (GB) have not yet been reported. In this study, we attempted to investigate the effect of EGB on liver damage in mice treated with EtOH for a short period by measuring the biochemical parameters in sera and liver tissues, and compared the hepatoprotective activity of EGB with that of GB. In addition, a UPLC-QTOF/MS method was developed for the identification of the major ginsenosides produced. Chemical profiling of EGB and GB was established, and in combination with quantitative analysis, was used for the systematic comparison of active compounds in EGB and the GB to understand the beneficial properties of major ginsenosides.

## 2. Results and Discussion

### 2.1. Identification of Standard Compounds in EGB

**Figure 2:**
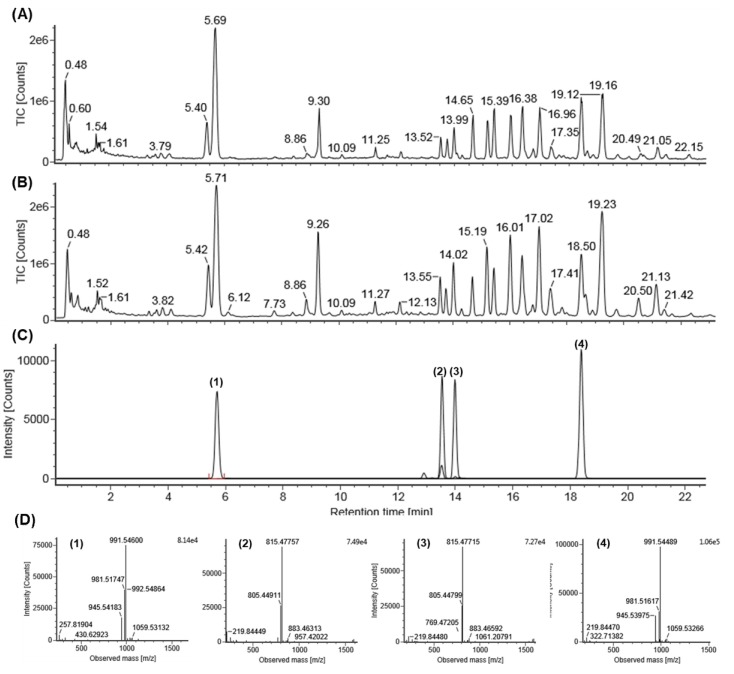
Total ion chromatogram (TIC) of EGB (**A**), non-enzyme-treated ginseng berry extract (GB) (**B**), and four standard analytes (**C**) by UPLC-QTOF/MS in negative-ion mode by selected ion monitoring, and representative QTOF/MS chromatograms (**D**) for ginsenoside Re (1), ginsenoside F5 (2), ginsenoside F3 (3), and ginsenoside Rd (4).

Standard compounds (1–4) were purified from the berries of *P. ginseng* by a series of chromatography procedures in our laboratory, and their structures were decided by a comparison of spectroscopic data (Mass, ^1^H-NMR, and ^13^C-NMR) with the literature data for ginsenoside Re (1), ginsenoside F5 (2), ginsenoside F3 (3), and ginsenoside Rd (4) [24,25,26]. The purity of the four ginsenosides was determined to be more than 95–99% by the normalization of the peak areas detected by UPLC analysis. For the high-throughput and sensitive analysis of various metabolites in ginseng berry, it was necessary to construct a robust method of profiling. The UPLC system, with its small particle size column, enables fast and effective separation of various ginsenosides. Thus, in this study, UPLC-QTOF/MS was applied to profile EGB and GB metabolites. For the quality control sample analysis, a standard of Ginsenoside Re was injected after every analysis of five samples. The precision of LC/MS analysis was determined by a coefficient of variation (CV) for RT and peak intensity. Figure 2 shows a typical total ion chromatogram (TIC) of the identified ginsenosides (1–4) with mass detection.

### 2.2. Quantitative Analysis of EGB by UPLC-QTOF/MS

Linear calibration curves were obtained for four ginsenosides (1–4) at different concentration levels. The calculated values of the calibration plots are summarized in Table 1. As shown in the table, the four ginsenosides (1–4) show excellent correlation coefficients. Detector counts (relative peak area) were linearly dependent on sample concentration over the range of 0.5–60 μg/mL for 1, 0.01–10 μg/mL for 2, 1–10 mg/mL for 3, and 1–20 mg/mL for 4. The limit of detection (LOD) of ginsenoside Re, F5, F3, and Rd were 0.010, 0.015, 0.004, and 0.001 ppm, respectively. The limit of quantification (LOQ) of ginsenoside Re, F5, F3, and Rd were determined to be 0.034, 0.049, 0.012, and 0.003 ppm, respectively, by UPLC-QTOF/MS in –ESI mode. The amount of ginsenoside Re, F5, F3, and Rd in the EGB obtained using validation methods (Table 1) was 45.9, 3.3, 4.0, and 6.2 mg/g, respectively.

### 2.3. Protective Effect of EGB on Serum AST and ALT in AILD

**Figure 3:**
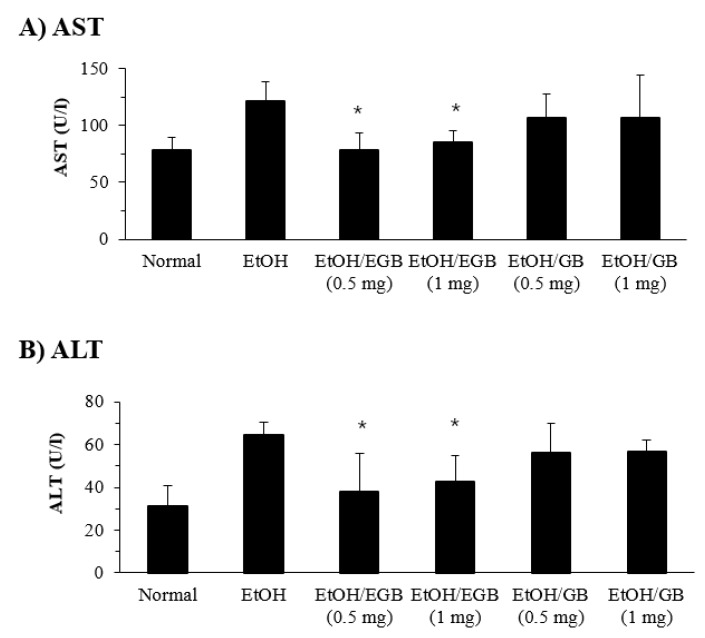
Effect of EGB on serum aspartate aminotransferase (AST) and alanine aminotransferase (ALT) in alcohol-induced liver damage (AILD). The AST (**A**) and ALT (**B**) levels were measured using serum specimens collected 1 day after the final administration of EtOH. * *p* < 0.05, compared with the EtOH-fed group.

Here, we used an intragastric feeding model for the AILD experiment. Mice were administered p.o. with EtOH to result in liver damage. As drinking is the major cause of alcohol delivery in humans, this experimental model is beneficial for examining the pathophysiological characters of AILD. In the course of liver damage caused by certain poisonous stimulations, the damaged hepatocytes secrete cellular enzymes such as AST and ALT into the bloodstream, and the concentration of these enzymes in the serum increases. Therefore, measuring the serum levels of AST and ALT is an effective tool for evaluating liver damage [27]. As shown in Figure 3, EtOH-fed mice showed marked increases in AST and ALT, which are indicative of liver damage. Treatment with EGB at a dose of 0.5 or 1 mg/mouse significantly suppressed the AST and ALT levels in the mice in AILD, although dose dependency was not recognized. On the other hand, treatment of GE caused a slight decrease in serum AST and ALT, but its inhibitory effect was not statistically significant. These results apparently suggest that oral administration of EGB can suppress liver damage induced by alcohol, and that its suppressive effect is higher than that of GB.

### 2.4. Antioxidant Effect of EGB in EtOH-Fed Mice

**Figure 4:**
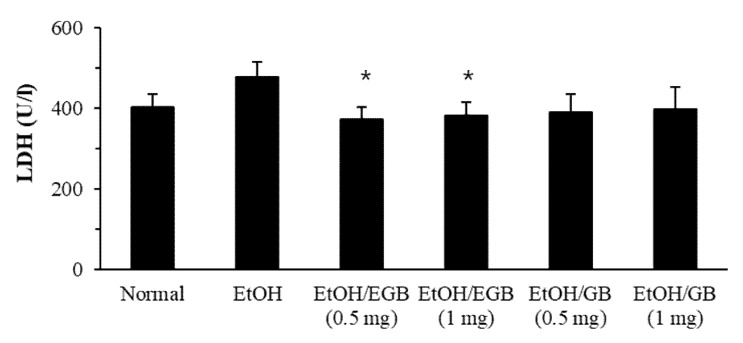
Effect of EGB on the level of serum lactate dehydrogenase (LDH) in AILD. The serum LDH level was measured using serum specimens collected 1 day after the final administration of EtOH. * *p* < 0.05, compared with the EtOH-fed group.

It is well known that AILD is basically associated with oxidative stress occurred by decreased antioxidant activities or increased free radicals, playing an important role in the pathogenesis of alcohol-induced hepatitis [28]. An increase in the level of serum enzymes, such as AST, ALT, and LDH, a biomarker of liver damage, is indicative of the dysfunction of hepatocytes and collapse of functional structure of cell membrane in the liver. Injury of liver tissue also results in the leaking of cellular enzymes of damaged hepatocytes into serum [29]. Superoxide dismutase is a pivotal enzyme having antioxidant activity via scavenging superoxide anion; catalase is an enzyme responsible for the detoxification of H_2_O_2_ generated by SOD, suppressing the formation of superoxide radicals [30]. Therefore, we tried to address the effect of EGB on AILD by investigating liver profile enzyme LDH and antioxidant enzymes such as SOD and catalase. The level of serum LDH increased in EtOH-fed mice, and the elevated LDH was significantly reduced by the oral administration of EGB at a dose of 0.5 and 1 mg/mouse (Figure 4). Non-enzyme-treated ginseng berry extract was also partially effective, but its activity was lower than that of GB.

**Figure 5:**
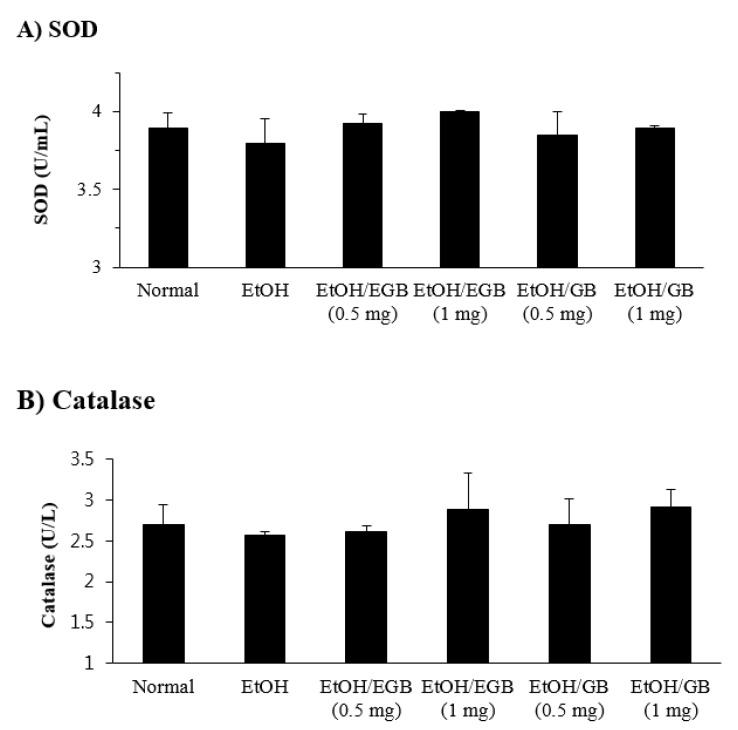
Effect of EGB on the level of antioxidant enzymes in liver tissues in AILD. The SOD (**A**) and catalase (**B**) levels were measured using the liver tissues collected 1 day after the final administration of EtOH.

**Figure 6:**
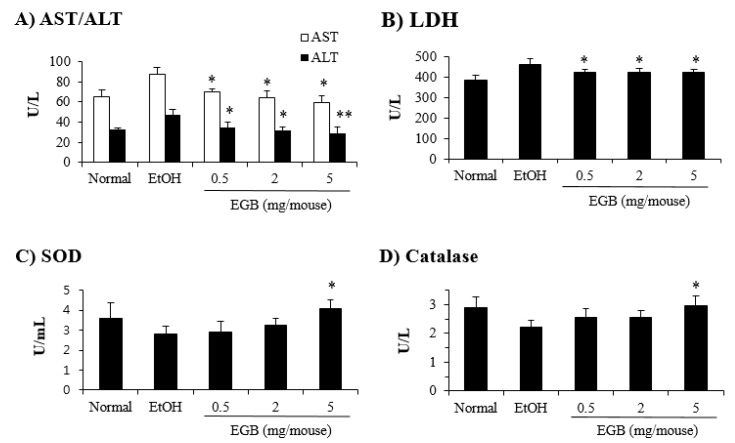
Dose-dependent effect of EGB on the expression of AILD-related enzymes. Mice were administered p.o. with the indicated doses of EGB, and the levels of liver profile enzymes, AST/ALT (**A**) and LDH (**B**) in the serum specimens and antioxidant enzymes such as SOD (**C**) and catalase (**D**) in the liver tissues were determined 1 day after the final administration of EtOH. * *p* < 0.05, ** *p* < 0.01, compared with the EtOH-fed group.

On the other hand, the levels of SOD and catalase in the liver tissues were reduced in EtOH-fed mice, and the decreased expression of these antioxidant enzymes was slightly recovered by EGB administration, although its enhancing effect was not statistically significant (Figure 5). In addition, when the effect of EGB on AILD was tested on a wide range of administration dosages (0.5–5 mg/mouse), a higher dosage of EGB significantly inhibited both liver profile enzymes (AST, ALT, and LDH), and antioxidant enzymes (SOD and catalase) in a dose-dependent manner (Figure 6). These results indicate that the inhibitory effect of EGB on AILD is caused by the suppression of liver profile enzymes (protection of liver cells against EtOH-induced damage), and enhancement of expression of antioxidant enzymes in the livers.

### 2.5. Changes in Organ Weight by EGB in EtOH-Fed Mice

**Figure 7:**
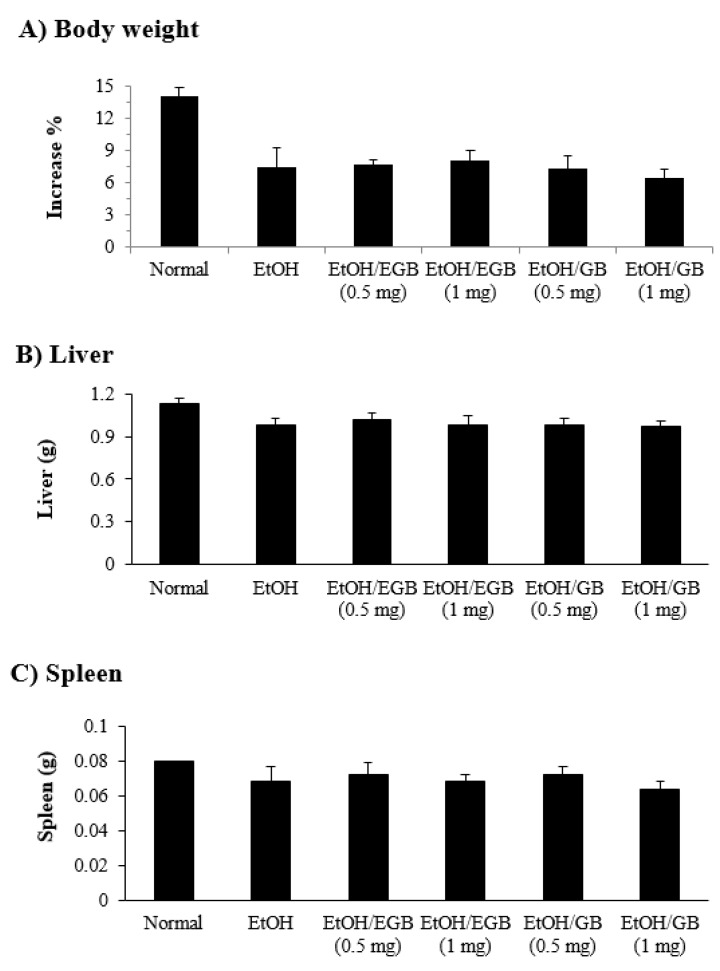
Effect of EGB on tissue weight in EtOH-fed mice. Mice were administered p.o. (per oral) with EtOH in the presence or absence of EGB. (**A**) Bodyweight; At the end of EtOH, livers (**B**) and spleens (**C**) were isolated and weighed. Bodyweight was expressed as an increase percentage of animal weight.

In this study, for induction of hepatic dysfunction, mice intragastrically received 25% EtOH for 7 days. At the end of the experiment, the liver and spleen weight, and bodyweight were measured. Mice fed with 25% EtOH showed reduction of not only bodyweight, but also liver and spleen weight (Figure 7); the reduction of organ weight could be attributed to the toxicity induced by EtOH exposure. Unfortunately, administration of EGB, which led to the significant reduction of EtOH-induced elevation of serum AST, ALT, and LDH, did not restore the loss of organ weights. The reason why EGB had no effect on the reduction of tissue weights is unclear; however, its effect may be associated with the nutritional support or protection of liver cells against alcohol-induced damage.

### 2.6. Anti-Inflammatory Effect of EGB on LPS-Stimulated RAW 264.7 Macrophages

**Figure 8:**
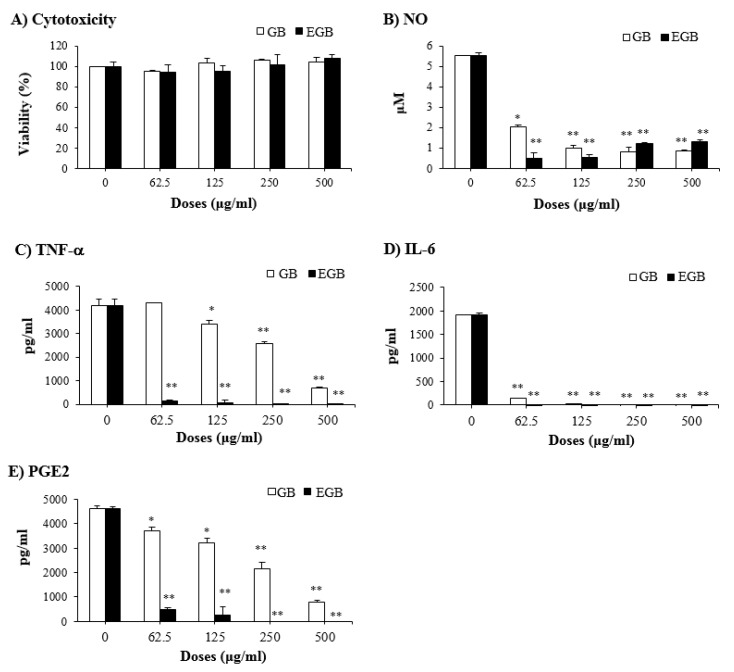
Anti-inflammatory effect of EGB in LPS-stimulated RAW 264.7 macrophages. The RAW 264.7 cells were pretreated with the indicated doses of EGB or GB for 15 h, and then stimulated with LPS (1 μg/mL) for 24 h. Cell viability (**A**) was determined by the MTT assay. The concentrations of NO (**B**), TNF-α (**C**), IL-6 (**D**), and PGE_2_ (**E**) in the culture supernatants were measured by commercial sandwich ELISA kits. * *p* < 0.01, ** *p* < 0.001, compared with the EtOH-fed group.

It is well recognized that acute alcohol consumption augments the level of circulating LPS in alcoholics with AILD [31]. The liver responses to gut-derived LPS in alcohol-induced injury, and, therefore, this is a critical step in the development of AILD [32]. Alcohol pre-exposed to the liver can prime Kupffer cells, the specialized macrophages in this tissue, and this primed hepatic cells’ response to LPS to be stimulated, resulting in an increased production of inflammation-related cytokines and mediators [33]. With regard to this, we investigated the inhibitory effect of EGB on inflammatory responses in LPS-stimulated RAW 264.7 macrophages. The MTT assay revealed that EGB treatment did not affect the toxicity against RAW 264.7 macrophage cells up to a concentration of 500 μg/mL (Figure 8A). Based on this result, the next experiments were carried out using EGB and GB with a maximum concentration of 500 μg/mL. Treatment with EGB significantly attenuated the production of inflammatory mediators such as NO, TNF-α, IL-6, and PGE2 in LPS-stimulated RAW 264.7 macrophage cells in a dose-dependent manner (Figure 8). Furthermore, similar to the results obtained in the AILD animal experiments, the inhibitory effect of EGB on LPS-induced inflammation in RAW 264.7 cells were higher than that of GB. The data clearly show that EGB has anti-inflammatory activity to ameliorate inflammatory responses in LPS-stimulated macrophages, and strongly suggest that EGB can suppress the production of inflammatory mediators released from Kupffer cells exposed to LPS during AILD progression. Taken together, this study implies that EGB has an inhibitory effect on AILD in mice via the suppression of liver profile enzymes (AST, ALT, and LDH), and enhancement of expression of antioxidant enzymes (SOD and catalase), and suggests the possibility that the hepatoprotective effect of EGB is also related to the inhibition of inflammation induced by LPS generated from the gut during AILD development. In this study, we demonstrate that EGB has a potential effect on AILD, and that EGB is a promising candidate for the development of natural product drugs or functional foods available for the prevention and treatment of AILD.

## 3. Materials and Methods

### 3.1. Preparation of Ginseng Berry Extract by Enzyme Treatment

**Figure 9:**
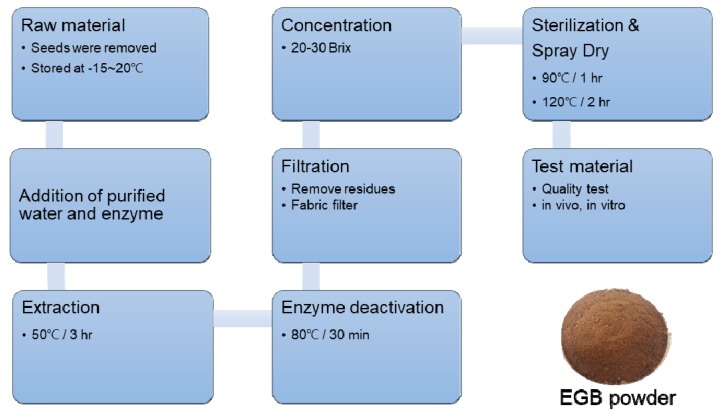
Manufacturing process for the production of enzyme-treated ginseng berry extract (EGB) powder.

Berries of six-year-old *P. ginseng* were obtained from the ginseng field at Hoengseong County, Gangwon Province (128°08′46.8″ E, 37°20′48.1″ N), according to the protocol of the “ginseng GAP standard cultivation guide line” developed by the Rural Development Administration (RDA), Republic of Korea [34]. A voucher specimen (NIHHS-201401S) was deposited at the Herbarium of the DHCR, National Institute of Horticultural and Herbal Science (NIHHS), RDA, Republic of Korea. The seeds were removed from ginseng berries, and the fruits were crushed and stored at −15~20 °C until extraction. Then, 1 L of purified water was added to equal amounts of powder, and 1.25 g of enzyme (Sumizyme SPC, Cas No. 9012-54-8, Shin Nihon Chemical, Anjo, Japan) was added to remove the cell wall of the plant. Ginseng berries were extracted with warm water at 50 °C for 3 h. After deactivation of the enzyme at 80 °C for 30 min, the extract was filtered through a fabric filter and residues were removed. It was then concentrated to a Brix of between 20 and 30 °C, and sterilized at 90 °C for one hour. The extract was spray-dried by spray dryer under 120 °C to obtain the final product, and was used for analyses and experiments (Figure 9).

### 3.2. Preparation of Sample and Standard Solutions

Standard solutions of ginsenoside Re (1), ginsenoside F5 (2), ginsenoside F3 (3), and ginsenoside Rd (4) were prepared by dissolving 1.00 mg each in 1 mL 80% methanol to yield a concentration of 1.00 mg/mL, and were kept at 4 °C. The standard solutions (1–4) were diluted with 100% methanol to obtain calibration solutions with ranges of 0.5–60, 0.01–10, 1–10, and 1–20 μg/mL, respectively. Finally, 1000 mg of EGB was accurately weighed and dissolved in fixed volumes (10 mL) of 80% methanol, filtered through a 0.20 mm filter paper, and kept at 4 °C.

### 3.3. Analysis of Ginsenosides Using UPLC-QTOF/MS

Ultra-high-performance liquid chromatography (UPLC) was performed using a Waters ACQUITY H-Class (Waters Corp., Milford, MA, USA) with an ACQUITY BEH-C18 column (1.7 μm, 2.1 × 100 mm). The mobile phases consisted of H_2_O with 0.1% formic acid (*v*/*v*), and acetonitrile (MeCN) with 0.1% formic acid (*v*/*v*). The elution gradient was as follows: 0–1 min, B 10–20%; 1–6 min, B 20–20%; 6–13 min, B 20–30%; 13–23 min, B 30–60%; and 23–25 min, B 60–95%. The injection volume was 2.0 μL and flow rate was 0.5 mL/min for each run. Next, high- resolution mass analysis was performed using Xevo (G2-S) quadrupole time-of-flight mass spectrometry (Q-TOF/MS, Waters Corp.) performing in –ESI mode (negative ion). Accurate mass measurement was obtained by means of an automated calibration system containing Leucine enkephalin as an internal reference (*m*/*z* 554.2615, –ESI mode). Optimal operating parameters were set as shown in Table 2.

### 3.4. Animals

The BALB/c mice (5 weeks old, male) obtained from Raon Bio (Yongin, Korea) were acclimatized for 1 week before the experiment. Animal experiments were performed in accordance with the guidelines of the Institutional Animal Care and Use Committee of Konyang University (Approval No. P-18-07-A-01).

### 3.5. Induction of AILD and Measurement of Biochemical Parameters

The animal experiment for AILD was performed as described previously [35]. Groups of seven BALB/c mice were treated with 25% EtOH (*w*/*v*, 5 g/kg bodyweight) (Merck Millipore, Billerica, CA, USA) via oral gavage for 7 days. Mice treated with EGB (EtOH/EGB) or GB (EtOH/GB) were administered p.o. with the indicated doses of each extract (0.5–5 mg/mouse) for 10 consecutive days beginning 3 days before EtOH treatment. The control (normal) mice received an equal volume of phosphate-buffered saline (PBS). One day after the final administration of EtOH, mice were euthanized, and blood samples and tissues were collected. The levels of serum alanine aminotransferase (ALT), aspartate aminotransferase (AST), and lactate dehydrogenase (LDH) were measured using DriChem 2500i (Fuji, Tokyo, Japan). Superoxide dismutase and catalase in the livers were determined by EnzyChrom assay kits (Fisher Scientific, San Francisco, CA, USA) according to the manufacturer’s instructions.

### 3.6. Cell Culture

A murine macrophage cell line RAW 264.7 was obtained from the Korean Cell Line Bank (Seoul, Korea). The RAW 264.7 cells were cultured in Dulbecco’s Modified Eagle Medium (DMEM) containing 10% FBS and antibiotics (100 U/mL penicillin and 100 μg/mL streptomycin) at 37 °C in 5% CO_2_ conditions.

### 3.7. Cell Viability

Viability of the cells was determined using MTT reagent (Duchefa, Haarlem, The Netherlands). The RAW 264.7 cells (1 × 10^4^ cells per well in a 96-well plate) were pretreated with EGB or GB at the indicated concentrations for 12 h, and then stimulated with LPS (100 ng/mL) for 24 h. The cells were added to 10 μL of MTT solution (0.5 mg/mL) and incubated for 2 h. After aspirating the supernatants of cell cultures, the formazan crystals were dissolved in 100 μL of dimethyl sulfoxide for 10 min. The optical density of each well was read at 540 nm in a microplate reader (Bio-Rad, Hercules, CA, USA).

### 3.8. Nitric Oxide, PGE2, and Cytokine Assays

The nitrite concentration in the cell culture supernatants was measured using a NO Detection Kit (iNtRON Biotechnology, Suwon, Korea), according to the manufacturer’s instructions. Cytokines (TNF–α and IL–6) and PGE2 in the cell culture supernatants were quantified by enzyme-linked immunosorbent assay (ELISA) kits (BD Science, San Diego, CA, USA) according to the manufacturer’s instructions.

### 3.9. Statistics

Results were expressed as mean ± standard error, and statistical significance was calculated using Student’s *t*-test.

## 4. Conclusions

This study showed that the oral administration of EGB significantly inhibited AST, ALT, and LDH level serum parameters indicating the severity of AILD, and enhanced the expression of antioxidant enzymes such as SOD and catalase in the liver tissues of EtOH-fed mice. In addition, treatment with EGB resulted in the significant inhibition of expression of inflammatory mediators like NO, TNF-α, IL-6, and PGF2 in LPS-stimulated RAW 264.7 macrophages. Collectively, EGB has a hepatoprotective activity to inhibit AILD, and its effect is related to the inhibition of oxidative stress and inflammation generated during AILD development. The advantages of UPLC-Q/TOF mass spectrometry include not only sensitivity and quality of detection capability, but also accurate measurement, making the structure elucidations easier. It can be used for the quantitative and qualitative determination of bioactive compounds, which is helpful in improving the QC of ethanolic extract from ginseng berry and its pharmaceutical preparations.

## Figures and Tables

**Table 1:** Linear regression data and contents of the validated method for the investigated compounds (1–4) in EGB ^a^.

Compounds	Rt ^b^ (min)	Calibration Curve ^c^	R^2^	Line Arrangement (μg/mL)	LOD (ppm)	LOQ (ppm)	Amount (mg/g)
**1**	5.69	*y* = 1333.7*x* + 5378.3	0.998	0.5–60	0.010	0.034	45.9
**2**	13.52	*y* = 1137.3*x* + 251.22	0.998	0.01–10	0.015	0.049	3.3
**3**	13.99	*y* = 1137.3*x* + 251.22	0.999	1–10	0.004	0.012	4.0
**4**	19.12	*y* = 3295.6*x* + 10613	0.998	1–20	0.001	0.003	6.2

^a^ Mean values of samples (*n* = 3). ^b^ Rt, retention time. ^c^ *y*, logarithmic value of peak area; *x*, logarithmic value of amount injected.

**Table 2:** Optimal conditions for Q-TOF/MS analysis of EGB.

Optimal Q-TOF/MS Condition	
Ion Source	ESI Negative Mode
Source Temp. and Desolvation Temp.	120 °C/550 °C
Cone Gas and Desolvation Gas Flow	30 L/h/800 L/h
Capillary and Cone Volt	3 kV/40 V
Mass Range (*m/z*)	100 to 1500
Collision Energy Range	20 to 45 eV

## References

[1] Frazier T.H., Stocker A.M., Kershner N.A., Marsano L.S., McClain C.J. (2011). Treatment of alcoholic liver disease. Ther. Adv. Gastroenterol..

[2] Gramenzi A., Caputo F., Biselli M., Kuria F., Loggi E., Andreone P., Bernardi M. (2006). Review article: Alcoholic liver diseased pathophysiological aspects and risk factors. Aliment. Pharmacol. Ther..

[3] Kaneko H., Nakanishi K. (2004). Proof of the mysterious efficacy of ginseng: Basic and clinical trials: Clinical effects of medical ginseng, Korean red ginseng: Specifically, its anti-stress action for prevention of disease. J. Pharmacol. Sci..

[4] Walsh K., Alexander G. (2000). Alcoholic liver disease. Postgrad. Med. J..

[5] Lieber C.S. (1988). Biochemical and molecular basis of alcohol-induced injury to liver and other tissues. N. Engl. J. Med..

[6] Purohit V., Gao B., Song B.J. (2009). Molecular mechanisms of alcoholic fatty liver. Alcohol. Clin. Exp. Res..

[7] Das S.K., Nayak P., Vasudevan D.M. (2003). Biochemical markers for alcohol consumption. Indian J. Clin. Biochem..

[8] Choi K.T. (2008). Botanical characteristics, pharmacological effects and medicinal components of Korean *Panax ginseng* C.A. Meyer. Acta Pharmacol. Sin..

[9] Lee C.H., Kim J.H. (2014). A review on the medicinal potentials of ginseng and ginsenosides on cardiovascular diseases. J. Ginseng Res..

[10] Li K.K., Gong X.J. (2015). A review on the medicinal potentials of *Panax ginseng* saponins in diabetes mellitus. RSC Adv..

[11] Park J.D., Rhee D.K., Lee Y.H. (2005). Biological activities and chemistry of saponins from *Panax ginseng* C.A. Meyer. Phytochem. Rev..

[12] Kim Y.K., Yoo D.S., Xu H., Park N.I., Kim H.H. (2009). Ginsenoside content of berries and roots of three typical Korean ginseng (*Panax ginseng*) cultivars. Nat. Prod. Commun..

[13] Wang C.Z., Wu J.A., Mcentee E., Yuan C.S. (2006). Saponins composition in American ginseng leaf and berry assayed by high-performance liquid chromatography. J. Agric. Food Chem..

[14] Qi L.W., Wang C.Z., Yuan C.S. (2011). Isolation and analysis of ginseng: Advances and challenges. Nat. Prod. Rep..

[15] Zhang Y.X., Wang L., Xiao E.L., Li S.J., Chen J.J., Gao B., Min G.N., Wang J.P., Wu Y.J. (2013). Ginsenoside-Rd exhibits anti-inflammatory activities through elevation of antioxidant enzyme activities and inhibition of JNK and ERK activation in vivo. Int. Immunopharmacol..

[16] Yoshikawa M., Morikawa T., Kashima Y., Ninomiya K., Matsuda H. (2003). Structures of New Dammarane-Type Triterpene Saponins from the Flower Buds of *Panax notoginseng* and Hepatoprotective Effects of Principal Ginseng Saponins. J. Nat. Prod..

[17] Lia P., Lva B., Jianga X., Wanga T., Ma X., Chang N., Wang X., Gao X. (2016). Identification of NF-κB inhibitors following Shenfu injection and bioactivity-integrated UPLC/Q-TOF-MS and screening for related anti-inflammatory targets in vitro and in silico. J. Ethnopharmacol..

[18] Tung N.H., Song G.Y., Woo S.H., Hyun J.W., Koh Y.S., Kang H.K., Shoyama Y., Kim Y.H. (2012). Ginsenosides from the Leaves and Flower Buds of *Panax ginseng* and their Pharmacological Effects. Curr. Bioact. Compd..

[19] Joo K.M., Lee J.H., Jeon H.Y., Park C.W., Hong D.K. (2010). Pharmacokinetic study of ginsenoside Re with pure ginsenoside Re and ginseng berry extracts in mouse using ultra performance liquid chromatography/mass spectrometric method. J. Pharm. Biomed. Anal..

[20] Lee H.J., Suh H.J., Lee H.S. (2010). A study on the utilization of enzyme treated ginseng leaf for cosmeceutical ingredient. Asian J. Beauty Cosmetol..

[21] Kim Y.C., Cho J.W., Lee Y.K., Yoo K.M., Rho J.H. (2007). Antioxidant Activity of Ginseng Extracts Prepared by Enzyme and Heat Treatment. J. Korean Soc. Food Sci. Nutr..

[22] Rae S.H., Lee H.S., Kim M.R., Kim S.Y., Kim J.M., Suh H.J. (2011). Changes of ginsenoside content by mushroom mycelial fermentation in Red ginseng extract. J. Ginseng Res..

[23] Im K.S., Chang E.H., Je N.G. (1995). A modified alkaline hydrolysis of total ginsenosides yielding genuine aglycones and prosapogenol. Arch. Pharm. Res..

[24] Teng R., Li H., Chen J., Wang D., He Y., Yang C. (2002). Complete assignments of 1H and 13C NMR data for nine protopanaxatriol glycosides. Magn. Reson. Chem..

[25] Li K.-K., Xu F., Gong X.-J. (2016). Isolation, purification and quantification of ginsenoside F5 and F3 isomeric compounds from crude extracts of flower buds of *Panax ginseng*. Molecules.

[26] Cho J.G., Lee M.K., Lee J.W., Park H.J., Lee D.Y., Lee Y.H., Yang D.C., Baek N.I. (2010). Physicochemical characterization and NMR assignments of ginsenosides Rb1, Rb2, Rc, and Rd isolated from *Panax ginseng*. J. Ginseng Res..

[27] Lim J.D., Lee S.R., Kim T., Jang S.A., Kang S.C., Koo H.J., Sohn E., Bak J.P., Namkoong S., Kim H.K. (2015). Fucoidan from *Fucus vesiculosus* Protects against Alcohol-Induced Liver Damage by Modulating Inflammatory Mediators in Mice and HepG2 Cells. Mar. Drugs.

[28] Checha F., Kaplowitz N. (1997). Oxidative stress and alcoholic liver disease. Alcohol Health Res. World.

[29] Nikam P., Nikam S., Sontakke A., Khanwelkar C. (2009). Biochemical Changes in Alcoholic Hepatitis with Phyllanthus Amarus Therapy: A Study. Biomed. Res..

[30] Wendel A., Jokby W.B. (1980). Glutathione peroxidise. Enzymatic Basis of Detoxification.

[31] Bode C., Kugler V., Bode J.C. (1987). Endotoxemia in patients with alcoholic and non-alcoholic cirrhosis and in subjects with no evidence of chronic liver disease following acute alcohol excess. J. Hepatol..

[32] Rao R. (2009). Endotoxemia and gut barrier dysfunction in alcoholic liver disease. Hepatology.

[33] Szabo G., Bala S. (2010). Alcoholic liver disease and the gut-liver axis. World J. Gastroenterol..

[34] (2009). Ginseng GAP Standard Cultivation Guideline.

[35] Harrison-Findik D.D., Schafer D., Klein E., Timchenko N.A., Kulaksiz H., Clemens D., Fein E., Andriopoulos B., Pantopoulos K., Gollan J. (2006). Alcohol metabolism-mediated oxidative stress down-regulates hepcidin transcription and leads to increased duodenal iron transporter expression. J. Biol. Chem..

